# Culturally informed views on cancer screening: a qualitative research study of the differences between older and younger Somali immigrant women

**DOI:** 10.1186/1471-2458-14-1188

**Published:** 2014-11-20

**Authors:** Nancy C Raymond, Warfa Osman, Jennifer M O’Brien, Nora Ali, Farnaaz Kia, Fardowsa Mohamed, Abdifatah Mohamed, Kathryn B Goldade, Rebekah Pratt, Kolawole Okuyemi

**Affiliations:** Department of Psychiatry and Deborah E. Powell Center for Women’s Health, University of Minnesota Medical School, Minneapolis, USA; New American Community Services, St. Paul, USA; University of Minnesota Medical School, Minneapolis, USA; Independent Research Consultant, St. Paul, USA; Department of Family Medicine and Community Health, University of Minnesota Medical School, Minneapolis, USA

**Keywords:** Immigrant health, Breast and cervical cancer screening, Somali, Barriers to screening

## Abstract

**Background:**

Somali women are infrequently screened for breast or cervical cancer, and there is a paucity of evidence-based interventions to increase cancer screening in this community. In order to create a culturally relevant intervention for Somali women living in Minnesota, we sought to understand what Somali immigrant women know about breast and cervical cancer, what are the attitudes toward screening and what cultural barriers are there to screen as well as cultural factors that would facilitate screening.

**Methods:**

In partnership with a community-based organization, New American Community Services (NACS), focus groups were conducted to explore the issues described above. Two focus groups were held with younger women age 20 to 35 and two were held with women age 36 to 65.

**Results:**

Twenty-nine women participated in the four focus groups. The women identified 1) differences in health care seeking behavior in Somalia verses the United States; 2) cultural understanding of cancer and disease; 3) barriers to mammogram or Pap screening; 4) facilitators to seeking preventive cancer screening; and 5) risk factors for developing cancer.

**Conclusions:**

Cultural misperceptions and attitudes need to be addressed in developing culturally-appropriate interventions to improve screening uptake for Somali women. A nuanced response is required to address barriers specific to younger and older groups. Culturally informed beliefs can be integrated into intervention development, preventive care and screening promotion.

## Background

Women who immigrated to the United States within the past ten years are less likely to be screened by mammogram or Papanicolaou (Pap) test than any other population group, including the uninsured [1]. In one study, Somali women were less likely to be screened for breast and cervical cancer than Cambodian and Vietnamese women [2], and it has been reported that of all African immigrants in the United States, Somali refugees and immigrants were the least likely to have received a Pap smear [3]. In a recent study of African immigrants in Minnesota, only 52% for cervical cancer and 61% of age eligible women had been ever been screened for breast cancer [4]. National data from the Centers for Disease Control indicates that 72.8% of White women and 77.9% of Black or African American women received Pap smears in 2010 [5]. The CDC data also states that in 2010 67.4% of White women and 67.9% of Black or African American women received a mammogram within the past 2 years [6]. Harcourt et al. did not ask about receiving a mammogram in the past 2 years [4]. In this paper we present findings from a study which aimed to bridge the gap in cancer screening and provide an understanding of the barriers to and perceptions of breast and cervical cancer screenings among Somali women living in Minnesota.

In the limited studies of the attitudes, perceptions, and barriers to screening among Somali women, culturally-specific nuances appear. The Somali language was only recently transcribed (in 1972) [7], and literacy rates remain low [7]. Health literacy can prove a more substantial barrier [8] as translations for medical terms are often poorly understood or simply do not exist [9]. Lack of knowledge about cancer and cancer screening processes is frequently cited among as a barrier to screening [10, 11], as well as low cancer risk perception [12, 13]. Moreover, fear and stigma surrounding cancer itself can influence screening decisions [14]. Religion also plays a role in the cultural context. Over 98% of Somalis are Sunni Muslims [9], and some interpret cancer as God’s will [11]. Additionally, religion influences a strong preference for female providers [15], and religious concordance when possible [11]. In the context of cervical cancer screening, female circumcision can represent a barrier to screening in the Somali community [10, 16]. Past studies have reported that women who had undergone female circumcision raised concerns about pain and provider perception during Pap smears [7, 17, 18].

The Somali community is subject to the more general barriers to access that hamper minorities, as well. As in many immigrant communities, the lack of regular access to a health care provider, health insurance, or low-income status can often represent a barrier to screening [19]. The experience with health care in the country of origin may be a factor [20], as many immigrant communities may view health care as curative rather than preventive [21]. Other barriers may include misconceptions about cancer overall [19], a negative personal attitude towards screenings, cultural beliefs and a lack of awareness of options [16].

Our research team sought to deepen our understanding of the knowledge and attitudes about cancer and preventive screening held by Somali women in our community. This information would serve as a basis for culturally adapting an evidenced-based intervention to increase breast and cervical cancer screening and thereby reduce health disparities in our Somali immigrant community. Our research added to the existing knowledge on this topic by focusing on the differences in the knowledge, attitudes and beliefs of younger and older women in the Somali community in Minnesota. We also, utilized a community assets-based approach and focused on aspects of the Somali culture that would facilitate preventive cancer screenings as well as the barrier to screening.

Minnesota has the largest Somali immigrant community outside of Somalia and Kenya. According to the U.S. Census Bureau, there are 33,404 Somalis estimated to live in Minnesota, mostly concentrated in the Twin Cities Metro area (2007–2011, American Community Survey 5 year estimates). Unofficial estimates are up to 70,000 primarily due to secondary immigration of Somalis from other states in the United States to Minnesota. The large number of Somali immigrants living in Minnesota and the low screening rates among this population makes Minnesota an ideal location to research the barriers to uptake of cancer screening for Somali immigrants and refugees.

The data below was collected through the implementation of focus groups. The specific aims of these focus groups were to explore the following questions:

1. What do women in the Somali community know about breast and cervical cancer, the causes and treatment?
2. What are the attitudes toward preventive health screenings?
3. What specific cultural barriers may prevent women from seeking mammogram and Pap smear?
4. Are there aspects of the Somali culture that facilitate breast and cervical cancer screening? If so, what are they?

## Methods

The University of Minnesota research team (NCR, JB, NA, KG, KO) partnered with a community-based organization called New American Community Services (NACS) (WO, FM, AM) to design and implement a community needs assessment that would provide information on how to increase breast and cervical cancer screening in the Somali immigrant community. NACS is a Somali run organization that connected community members to economic, housing and health care services and offered English as a second language courses. NACS had previous experience in doing community needs assessments around health care issues prior to partnering with us on this project. NACS meet with and conducted key informant interviews to find out what Somali providers saw as the salient issues regarding breast and cervical cancer screening in their community. Collectively, the community/university team used data from these interviews and to design a moderator’s guide and implemented focus groups to answer the research questions identified above and described below in detail. These methodologies were ones that NACS had used in previous programs and they had worked well in their community.

### Development of the focus group moderator’s guide

Questions for the focus group moderator’s guide were developed based on discussions as a research team, and reflection on findings from initial key informant interviews, which are not reported here, but played an important role in the development of the moderator’s guide. The key informant interviews were conducted with ten Somali community leaders, five men and five women.

### Recruitment of focus group participants

Two Somali community health educators (CHE), who worked for the community- based partner organization, invited self-identified Somali women who had immigrated to Minnesota from East Africa to participate in a focus group. Some of the participants were associated with the partner organization. Others were personal contacts of the CHEs. All recruitment was done through conversations with potential participants. Once recruited some of the participants asked if they could invite a friend to participate in a focus group. We accepted these participants into the group. All women who were willing to participate were included in focus groups.

The community/university research team anticipated that younger Somali women might have different perspectives on cancer screening than older Somali women, thus we purposefully sampled for younger Somali (20–35) and older Somali (36–65) women. The age of 35 was suggested by the community partners as an appropriate age to distinguish younger and older women in the community. Lastly, having focus groups for older and younger women was appropriate because breast cancer screening is recommended only for women over 40, while cervical cancer screening should begin by 21 years of age. Participants were monetarily compensated $30.00 for participation in the focus group. The University of Minnesota’s Institutional Review Board approved and monitored this study. The study adhered to the RATS guidelines on qualitative research [22].

### Focus group procedures

Focus groups were led by a medical anthropologist (KG) or two bilingual (English and Somali) community health educators (CHE) who were trained in qualitative research methods by the medical anthropologist (KG). All participants were asked to complete a demographic survey designed by the research team; however the older women declined to complete them. Given the sensitivity of the topic and the commonplace reluctance of this group to provide information to institutions (such as the University), participants were not excluded if they did not provide this information.

The younger women’s focus groups were conducted in English and the older women’s focus groups were conducted in Somali. The moderator’s guide included semi-structured questions in the following domains: 1) Views on health care; 2) Knowledge of cancer; 3) Perceived risk of cancer; 4) Preventing cancer; 5) Barrier to screening: Circumcision; 6) Barrier to screening: Virginity; 7) Facilitators to screening. All focus groups were audio-recorded. The English focus groups were transcribed by trained student research assistants. The Somali focus groups were simultaneously translated and transcribed by the bilingual CHEs.

### Qualitative analysis of the focus group data

Focus group data were analyzed by the university/community research team (n = 8) during multiple group meetings. The approach to analysis drew on the Immersion Crystallization Method [23], which allows for the systematic review of transcripts to identify themes. Each member of the research group reviewed the first transcript and identified themes. Then the researchers met as a group to arrive at agreement consensus on the major themes emerging from the first transcript. Two bilingual CHEs and one English speaking master’s prepared CHE went through the transcripts individually to code the parts of the transcripts that that supported each of the major themes. All of the subsequent transcripts were coded with these initial themes and if other themes were identified during the reviews then these were brought to the research team for discussion. The entire team then met to discuss the analysis and to reach agreement on what best represented the community’s perspective regarding what was needed to develop culturally relevant interventions to increase breast and cervical cancer screening in the Somali community.

## Results

### Participants

Twenty-nine women from the Somali community participated in four focus groups; two groups were with younger women and the other two with older women. While the size of the focus groups were smaller than planned the smaller group size may have been more appropriate given the sensitive nature of the discussions. All participants were taken through a consent process where information was provided in both verbal and written formats. Consent forms were available in English and Somali. All participants gave written informed consent at the end of the consenting process. The older women declined to complete the demographic survey and the younger women did not fully complete the questionnaire. Therefore our demographic data is limited. All the women identified as Somali (100%), and over half of them were not yet married (57%). The younger women had been living in the United States from as little as 5 years to as much as 15 years. Our knowledge of the older women’s demographic information is limited, as all participants declined the demographic survey. From discussions with our community-based organization partners who led the focus groups and have personal knowledge of the participants, we know they were all Somali, and all over 40.

### Themes identified through the focus group analysis

There were five themes identified across the focus groups, and these were 1) differences in health care seeking behavior in Somalia verses the United States; 2) cultural understanding of cancer and disease; 3) barriers to mammogram or Pap screening; 4) facilitators to seeking preventive cancer screening; 5) risk factors for developing cancer. We will now discuss those in turn.

### Differences in health care seeking behavior in Somalia verses the United States

Women stated that in Somalia the doctors understood them from both a cultural and linguistic perspective while in the U.S. they must rely on interpreters. Both younger and older women saw major disadvantages to receiving care of any type in the U.S. because of potential misunderstandings between doctors and patients due to the necessity of using translators to communicate.

*It's really hard for someone to explain to the doctor exactly what's wrong with them. I mean, we use a lot of like, cultural words to describe what's wrong with you, where it hurts, and some of the doctors don't understand that and even some of the translators don't know how to translate those things. [Focus Group 1, younger]*

Some older women commented on the communication difficulties leading to lack of trust or understanding about their needs in the health care system in the United States:

*The other thing is, there is lack of trust between the system and us because we don't [have] the same culture. In Somalia, doctors knew more about our "background" - they knew everything about us, the illness, the environment, the food we eat but these doctors are different, they don't know me. They don't know our history, family, culture, what the Somali people use and that is the root cause of the problem. [Focus Group 4, older]*

Older women were more likely to state that they only go to the doctor when they are sick. They described that in Somalia they waited in line to see a doctor when they were ill. They recognized that the practices were different in Minnesota, in part due to a perception that people get sick more often here. Younger Somali women acknowledged that in Somalia, it was custom to go to the doctor only when sick, however the participants described developing the practice of going for routine preventive care for themselves and their children.

*And generations are changing…We’ve been um, educated enough or accustomed enough to get our annual exams and take your kids for check ins, and um, it’s just part of our culture now. Where, I think maybe, in the past, like, 10 years ago, 15 years ago, that wasn’t the case for our community. But I think it’s very much changing. [Focus Group 1, younger]*

This disconnect between health care practices in Somalia, and health care practices in the United States appeared to lead to a general distrust of doctors, and a fear among older women that American physicians were more likely to make mistakes.

*Quick decisions are often made about immigrant patients by the healthcare providers. Our doctors in Somalia used to take time to make the decisions about their patients. They were not rushing to perform surgery or prescribing the wrong medication or just destroying patients’ body parts that they will regret later. So, all these problems that I mentioned bring the differences between our country and this country. [Focus Group 4, older]*

As a result these many different ways the US health system was perceived, women were reluctant to seek screening in general, or any other type of health care in the U.S. unless they were actively symptomatic.

### Cultural understanding of cancer and disease

Both the younger and older Somali women participants commented that cancer was virtually an unknown diagnosis before they came to the United States. Participants remarked that there is no word for “cancer” in Somali, and that cancer was not a well-known disease in Somalia.

*Breast and cervical cancer, they aren’t really talked about much. In our community, it’s like, when people used to get sick back home, they didn’t identify it as cancer… It was always something else that they, you know, died of, or was diagnosed. [Focus Group 1, younger]*

Cancer was not only described as being rare, but also as stigmatized. Younger women remarked that cancer evoked secrecy and shame; in some cases being associated with other stigmatized illnesses, like HIV. Shame and fear of certain death from cancer led women to avoid the doctor.

*People didn’t think it was something preventable, you know, that you can live free of cancer. People automatically believe that you’ll die from it. So if you’re dying anyways, why bring, you know, a bad name to your family and make them feel associated with the disease. [Focus Group 1, younger]*

While both younger and older women talked about the secrecy, shame and stigma of cancer, the primary role of God’s will and fate in health, was predominantly mentioned by older women in this sample. There was also the view that only God could cure cancer.

*By culture we are Muslim people and we believe you can get cancer by God's will. When this killer disease kills someone, we believe that that person's time has expired and it is time to go. As Muslims we have our own beliefs and we believe that everything happens according to God's plan. You will have good health when God grants to you and you will die according to God's plan as well. No matter what treatment you get it will only works with God's approval. So we have faith in God. [Focus Group 4, older]*

Others expressed similar sentiments

*If a person dies I would hear it was because of God’s will, not cancer [Focus Group 3, older].*

*May God protect us from it because only God can protect, whether you go to the doctor or not. [Focus Group 3, older]*

*Cancer is a disease that is brought by God and it is a killer disease. Only God can cure it. [Focus Group 4, older]*

While older women emphasized the role of God and fate in disease more than younger women, younger women concurred that religion was a significant barrier to accepting the meaning of prevention.

*I believe that, well, our religion. I don’t think there’s such a thing as prevention. I mean, our religion says, if you’re meant to get it, you’re gonna get it. [Focus Group 1, younger]*

However overall, the influence of religion was notably less present in the focus groups with younger women. It was mentioned less often, and was not the same focus of discussion as it was with the older women. This does signal there may be a change in attitudes over time about the influence of religion in making decisions about screening.

### Barriers to mammogram or pap screening

Both younger and older women described concerns that radiation from the mammogram could actually cause cancer. As one of the older women stated,

*[Mammography] has radiation. I am one of the people who believe that the radiation that it produces actually causes [breast cancer]. [Focus Group 4, older]*

Younger women had more concerns about modesty and feelings of shyness than the older women. They also expressed concern about the pain caused by a mammogram more frequently than the older women.

There were many similarities between the younger and older Somali women on the perceived barriers for Somali women to have a Pap smear. As with the mammogram, all women agreed that issues around modesty and shyness were significant barriers. One of the older women expressed this sentiment with a reference to religion requiring modesty about the body.

*You are a Muslim person and shouldn’t be exposing your behind to people [Focus Group 3, older]*

One of the younger participants, however, did recognize that not getting a Pap smear as a result of this modesty and shyness may increase her risk of developing cancer.

*The Somali woman, she does not feel comfortable with others looking at her private body, unless she is having a baby. That is the only time she might allow the doctor to look at her private parts. That’s what causes us to have greater risk [for cancer]. [Focus Group 2, younger]*

Both younger and older women shared concerns about the logistics of the Pap smear as it relates to female circumcision and pain. The Pap smear was perceived to cause pain. Younger women also expressed concern that the Pap smear itself would jeopardize their culturally valued virginity.

*It’s like a taboo; you have to be a virgin before you get married, period. No Pap test or whatever. [Focus Group 2, younger]*

Older women didn’t think that the Pap smear would affect the virginity of younger women.

At the same time, younger women expressed concerns about maintaining a positive reputation in the community as a virgin. They feared that their virginity would be questioned if they were seen getting a pap test at a clinic, and that maintaining the perception of virginity outweighed the risks of not being screened for cancer.

*I don’t think they would understand. Like why would she need a Pap smear, if she’s never been married? And if you did then maybe you know, people might get the idea that you might not be a virgin, and you know, there’s reasons why you’re worried about getting your Pap smear. [Focus Group 1, younger]*

### Facilitators to seeking preventive cancer screening

Despite the barriers to screening, many women expressed openness to screening. Younger women in particular acknowledged that there has been a generational shift towards accepting screening and preventive care. Younger women also talked about the significance of the role of women in the Somali community. They indicated that the importance of women in the Somali culture could provide motivation for self-care and for encouraging other women to take advantage of preventive health screening.

*In the Somali culture, in our culture in general, we think, the female figure is very strong. Very strong. Whether she is a mother or an aunt, or it’s…they stand at a very higher, um, I mean, form of respect and honor in our community and culture. Therefore, having that family member always present really does help the community…once you’re saying certain illnesses are going to threaten them being there, present for the family or the community, that’s where the community would… be awoken to do something about it….Women are the drive for everything in the community basically…[Focus Group 1, younger]*

One of the older women spoke of the importance of staying healthy in order to fulfill the role of childbearing. The cultural significance of child-bearing was also referenced, and taking care of the female body was therefore an important and valued part of being able to successfully have a baby.

Older women confirmed that religion can be used to encourage health, even though the role of fate was strongly emphasized. As one woman stated, religion did not forbid preventive health care as such. While both younger and older women participants stated that they were more likely to see a doctor if there were problems, like pain or issues getting pregnant, they both expressed openness to the idea of getting checkups. Some participants in both age groups expressed the importance of regular health check and early identification of health problems.

### Risk factors for developing cancer

Many participants had correct information about factors that could increase a person’s risk of getting cancer. These included individual risk factors such as: heredity, smoking, using a sheesha (glass pipe) to smoke, excessive sun exposure and HPV. They also cited environmental risk factors such as secondary smoke and the environmental pollution in the US.

*…here I see there are environmental issues that cause it, chemical, uh, pesticides in the food, uh pollution in the air, you know, I heard some, scientific explanations like that. [Focus Group 1, younger]*

However there were also some misconceptions about the causes of gynecological cancers such as heavy bleeding after labor and bearing children in an unclean environment.

*It’s like, this lady come over, pushing your baby out. She never cleans the inside of your body, probably leaving more blood that can get stuck, and have a cancer. Finally you can die that way. [Focus Group 2, younger]*

Finally, there was discussion regarding factors that could protect you from having cancer such as: eating fresh foods, walking and vitamin D.

*…it has something to do with the environment you live in. I mean, my parents generation, where they’re used to all natural foods and vegetables, and I mean, going out, Vitamin D, and all that stuff… [Focus Group 2, younger]*

## Discussion

The purpose of this study was to gather knowledge to better understand what Somali immigrant women know about cancer, the acceptability of mammogram and Pap smear as screening modalities, and any age-based differences in attitudes toward screening, in order to create a culturally relevant intervention for Somali women living in Minnesota.

The data presented above are concordant with another recently published by our group [24]. This study reported on key informant interviews of women who were known to offer health-related guidance for member of the Somali community such as interpreter, community health workers and health care providers. The authors identified barriers to screening such as mistrust of the health care system based on reports by friend or relatives regarding problems they encountered, difficulty receiving health care because of language barriers, a preference for seeing providers with the same religious background and for seeing female providers, fear of pain and embarrassment, stigma related to a diagnosis of cancer, and accepting the will of God as the cause of health problems [24].

Many of the barriers identified in this study, such as a lack of accurate knowledge about breast and cervical cancer, are also reflected in the literature on Somali women [4, 10–12, 15]. Our findings indicated that there is considerable stigma surrounding a cancer diagnosis that leads to secrecy and shame. Both older and younger women expressed concerns about the radiation from mammograms, and the fear that mammograms cause cancer. This perspective is also reflected in the literature that health care is not seen as preventive [21], and that there was a lack of experience with cancer screenings in Somalia [20]. In our study, we identified that women had significant concerns about increased pain during a Pap smear due to their female circumcision [11]. Addressing these concerns in an intervention will allow for such views to be addressed and accurate information on cancer to be offered to participants.

This research adds to the literature on the differences between younger and older Somali women. While both groups shared some common concerns about access to health care or views on screening, it was clear that views were changing over time and younger Somali women had openness to preventive care that would help encourage screening uptake. However, there is a need for particular caution in addressing concerns about virginity with regard to the Pap smear for younger women, since lack of virginity compromises marriageability.

Another interesting difference from this research was the lessening influence of religion as a barrier to screening for younger women. In recent studies findings show that faith and religion play an important role in perceptions of health care in the Somali community. There is a strong belief in fate, and that cancer, if present, is God’s will [11, 12, 15]. Other studies have highlighted that there are several aspects of the Muslim faith that can be used to support screenings as well [20]. This includes requirements of honoring God through cleanliness and maintaining good health via physical exercise, taking medication as needed, and avoiding risky health habits. Furthermore, responsibility to family and oneself is realized by taking care of one’s health [10, 25]. Our research would suggest that incorporating these messages has particular importance for older Somali women.

Lack of access to culturally competent health care is one of the most significant barriers to reducing health disparities for minority populations [8, 18]. Differing communication styles between physicians and patients is an important barrier to proper health care access among immigrant and refugees in the United States [8, 26]. It has been recommended that health care providers take special care to show understanding and patience when treating Somali patients by expressing genuine desire to understand cultural differences, such as acknowledging preference for female physicians [18, 27, 28], and to ensure qualified interpreters are used in health care settings [9, 29].

### Limitations

This focus group study used a small group of women from one geographical area who may have self-selected into the study based on their interest and experience with cancer screening or breast and cervical cancer. Our study may not adequately reflect the views of all women in the community. The topics discussed were culturally sensitive and those who elected not to be a part of a discussion of such topics may have had very different opinions that those we have presented. Additionally, our data may not represent the views of Somali women who settled in other regions. Older participants declined to provide demographic information, which may limit generalizability; however, in our experience, declining the provision of this information can be commonplace when conducting community based research with minority communities on a sensitive topic.

## Conclusions

Our study showed that there are cultural misperceptions and attitudes which need to be addressed in developing culturally-appropriate interventions to improve breast and cervical cancer screening uptake for Somali women. One of the key messages that women in the Somali community need to receive is that cancer can be treated and can be curable if it is diagnosed early. Preventive screening is essential for early diagnosis prior to the onset of symptoms and can save lives.

A nuanced response is required to address barriers specific to younger and older groups of women. Explaining informed beliefs about many topics, including the importance of virginity, can be integrated into intervention development, preventive care and screening promotion. Providers need to be educated regarding how to respond to and work with women who have had “female circumcision” and taking into account the need for a smaller speculum. Same sex providers should be provided if possible. The community members told us that their culture values women. Therefore, providers can emphasize that screening is a way of supporting the health of the women in the community. Providers must also recognize that the Somali community is traditionally an oral culture and that information is spread by word of mouth. Successful experiences with the health care system, as well as perceived or actual medical mistakes, are shared by community members amongst each other. Making sure that patients understand the benefits, risks and side effects of procedures and treatments is essential to maintain a good relationship with the community members.
